# Low-Cycle Fatigue Behavior of 3D-Printed PLA Reinforced with Natural Filler

**DOI:** 10.3390/polym14071301

**Published:** 2022-03-23

**Authors:** Miroslav Müller, Vladimír Šleger, Viktor Kolář, Monika Hromasová, Dominik Piš, Rajesh Kumar Mishra

**Affiliations:** 1 Department of Material Science and Manufacturing Technology, Faculty of Engineering, Czech University of Life Sciences Prague, Kamycka 129, 165 00 Prague-Suchdol, Czech Republic; muller@tf.czu.cz (M.M.); pisd@tf.czu.cz (D.P.); mishrar@tf.czu.cz (R.K.M.); 2 Department of Mechanical Engineering, Faculty of Engineering, Czech University of Life Sciences Prague, Kamycka 129, 165 00 Prague-Suchdol, Czech Republic; sleger@tf.czu.cz; 3 Department of Electrical Engineering and Automation, Faculty of Engineering, Czech University of Life Sciences Prague, Kamycka 129, 165 00 Prague-Suchdol, Czech Republic; hromasova@tf.czu.cz

**Keywords:** additive manufacturing, 3D-printing, PLA polymer, biological filler, cyclic test, SEM

## Abstract

Additive production is currently perceived as an advanced technology, where intensive research is carried out in two basic directions—modifications of existing printing materials and the evaluation of mechanical properties depending on individual production parameters and the technology used. The current research is focused on the evaluation of the fatigue behavior of 3D-printed test specimens made of pure PLA and PLA reinforced with filler based on pinewood, bamboo, and cork using FDM (fused deposition modeling) technology. This research was carried out in response to the growing demand for filaments from biodegradable materials. This article describes the results of tensile fatigue tests and image analysis of the fracture surface determined by the SEM method. Biodegradable PLA-based materials have their limitations that influence their applicability in practice. One of these limitations is fatigue life, which is the cyclic load interval exceeding 50% of the tensile strength determined in a static test. Comparison of the cyclic fatigue test results for pure PLA and PLA reinforced with natural reinforcement, e.g., pinewood, bamboo, and cork, showed that, under the same loading conditions, the fatigue life of the 3D-printed specimens was similar, i.e., the filler did not reduce the material’s ability to respond to low-cycle fatigue. Cyclic testing did not have a significant effect on the change in tensile strength and associated durability during this loading interval for PLA-based materials reinforced with biological filler. Under cyclic loading, the visco-elastic behavior of the tested materials was found to increase with increasing values of cyclic loading of 30%, 50% and 70%, and the permanent deformation of the tested materials, i.e., viscoelastic behavior (creep), also increased. SEM analysis showed the presence of porosity, interlayer disturbances, and at the same time good interfacial compatibility of PLA with the biological filler.

## 1. Introduction

Current trends in globalized society are associated with environmental aspects that accompany human activity. An example is the implementation of environmental management systems (EMS) in manufacturing industries. However, even ordinary consumers currently prefer environmentally friendly products. When using 3D printing, the use of environmentally friendly filaments is preferred for products [1].

Additive production was originally limited, primarily, to product prototypes. However, now it is extensively used for the development of final products and their components [2]. Additive production using 3D printing technology involves applying material in layers, unlike conventional production such as welding, foundry, or machining [3]. The greatest advantage of 3D printing technology is the control of production and variability of the input material—the filament [3]. Commercial polymeric products are mainly manufactured by injection molding, blowing, or compression molding, especially in the case of large-scale industrial production. A production method using 3D printing technology eliminates the dependence on large industry. It allows the production of small series, and enables fast product modification, etc. [4]. There are many ways to print 3D products. One of the most common methods is FDM (fused deposition modeling) technology, which works with the use of a printing filament. The advantage of FDM is the adaptability to commercial filaments and their variability, versatility, and simplicity [5].

Quality 3D printing of parts (and, subsequently, their mechanical properties) affects various parameters that need to be examined [6]. Due to the dynamic growth of interest in a sustainable economy, research into biological materials and fillers is essential for additive technologies [7]. Biopolymers are an essential part of modern life because of their biodegradable nature. An important representative of biopolymers is, for example, polylactic acid (PLA), which is one of the most widely used commercially available materials [7]. It is also a significant printing material used in additive production using 3D printing [2]. PLA is a very widespread material, mainly due to its biodegradability [3]. The disadvantages of PLA include higher costs of material and some inferior mechanical properties compared to polymers such as polypropylene (PP) and polyvinyl chloride (PVC) [3]. As in the field of polymeric composite materials with a natural filler, the costs of this material and the mechanical properties can be substantially modified [5,8,9,10,11].

Research on mechanical properties in the field of 3D printing focuses on the evaluation of printing materials and individual aspects of production such as the influences of parameters including polymer orientation, thickness of layers, feed rate, raster angle, raster width, air gap, nozzle diameter, etc. on tensile strength and elongation [12,13,14,15,16]. Another important direction is research in the field of various modifiers and additives to the printing material, in which microparticles and nanoparticles were used [3,7,17,18,19]. Examples are cellulose, wood, cork, rice husks, flax, bamboo, and other plant-based fillers that can be added to a biodegradable thermoplastic material e.g., PLA, which is used in 3D printing by FDM (fused deposition modeling) [20,21,22,23,24,25,26]. Tianyun et al. [27] point out that strength analysis of multi-material products associated with printing strings is an important area of research.

Interest in the addition of natural fibers as fillers to conventional materials for additive production has increased in recent years, mainly due to the trend of sustainable development in the field of materials research. The benefits are not only in the field of sustainable product development, but are also related to a possible improvement in mechanical properties [20,28]. However, it is always necessary to test not only mechanical properties, but also the mutual interaction between the filler and the matrix e.g., by SEM analysis [29,30]. Research on the development of biodegradable additives for 3D printing is important for ensuring the sustainability and development of the industry and the minimization of synthetic materials [7,31]. There is a similar trend in the field of biological fillers used in polymer composite materials [10,32]. Wood flour is usually added to PLA for aesthetic reasons, but mainly to reduce costs (economic effect) and improve thermal properties. This is typically associated with the possibility of the further use of 3D printing technology [1]. The advantages of biological materials and the associated degradation should be taken into account, especially in the use of PLA with a relatively short life cycle and susceptibility to degradation processes [31]. The products developed should be stable in relation to fatigue behavior during their use.

Polymeric materials are usually subject to cyclic stress during operation, i.e., cyclic fatigue, which is more pronounced than metallic materials. Cyclic fatigue causes irreversible failure before reaching maximum strength [33]. The strength and durability of polymeric materials are reduced even at low stress levels due to cyclic fatigue [30,34,35]. This kind of cyclic fatigue of the material is the most destructive form of mechanical load. Cyclic loading is the most common cause of degradation of such materials in practice [33]. The strength of composite materials is affected not only by the reinforcing component used, but also by the transfer of stress between the individual reinforcements and the matrix. If the reinforcements are properly impregnated by the matrix, then the transfer of stress between the matrix and the reinforcement is efficient, which has a significant effect on the service life of the material [30]. Cyclic tests are essential for determining the suitability of material for practical applications [30,33,34].

From the results reported by Shahar et al. [36], it is clear that PLA reinforced with kenaf particles exhibits relatively good cycle fatigue properties in combination with an additive technology and reduced production costs. It was clear from the result that fatigue life increases with an increasing amount of filler in PLA. A secondary positive effect is the reduction in the cost of natural filler material compared to pure PLA. Azadi et al. stated that PLA samples have better fatigue life as compared to ABS samples. It was further pointed out that fatigue strength of samples developed by 3D printing in the horizontal direction is higher than the fatigue strength in the vertical direction [6]. The results of fatigue tests are of great importance for industry and the production environment as they lead to sustainable use of the given materials [28]. Gang et al. [37] stated that the loading rate and the course of loading will have a significant effect on the fatigue behavior of polymeric materials. For this reason, it is very important to obtain data on deformation and stress during the fatigue process.

This article presents the results of the static tensile test and fatigue behavior in cyclic tensile tests that simulate practical application due to long-term loading. The tested materials are FDM-printed test specimens made of pure PLA and PLA reinforced with natural particles based on pinewood, bamboo, and cork. The aim of the research is to determine the basic mechanical characteristics at constant 3D printing parameters in the tensile test and especially to investigate fatigue behavior at different cyclic stress intensities. The aim is to confirm whether the incorporation of biological fillers into PLA brings the expected mechanical benefits from a functional point of view. The impact of the tested natural fillers is also evaluated by comparing the results with unreinforced PLA specimens.

## 2. Materials and Methods

### 2.1. Materials and Preparation of Test Specimens

Polylactic acid (PLA)-based pure and reinforced samples with natural fillers were investigated in this research. The tested materials were in the form of filaments intended for the 3D printing of samples, which were obtained from the company PLASTIKA TRČEK (PLASTIKA TRČEK d.o.o., Ljubljana, Slovenia). It is necessary to define the basic mechanical properties of these materials.

The samples were prepared from pure PLA and PLA reinforced with natural particles based on pinewood (designated PLA PW), bamboo (designated PLA B) and cork (designated PLA C). PLA is a material that is environmentally friendly and can be broken down by microorganisms in nature [27]. This has led to the choice of material for cyclic loading test, which is important for practical applications.

**Figure 1:**
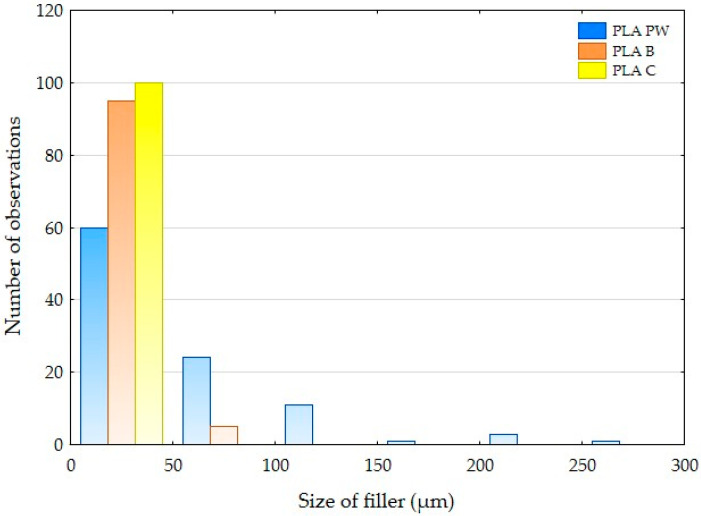
Histogram of PLA reinforced with natural reinforcement in the direction perpendicular to the cross section.

The particle size of the natural reinforcement in PLA was determined by optical analysis using the Gwyddion program (version 2.49, David Nečas and Petr Klapetek, Brno University of Technology, Brno, Czech Republic) and electron microscopy (SEM) images. The results of this image analysis are shown in Table 1 and summarized in the histogram in Figure 1. Table 1 shows the dimensions of the natural fillers measured perpendicular to each other and the particle length. From the presented dimensions it is clear that the fillers based on pinewood (filament designation PLA PW) have the largest dimensions. The results also show considerable dimensional variability in fillers B, PW and C, i.e., the filaments PLA C, PLA B and PLA PW contain large and small particles characterized in Table 1 and Figure 1.

The basic parameters of the tested material/filament and the pure PLA filament are given in Table 2 and are based on the recommendations by the manufacturer.

**Figure 2:**
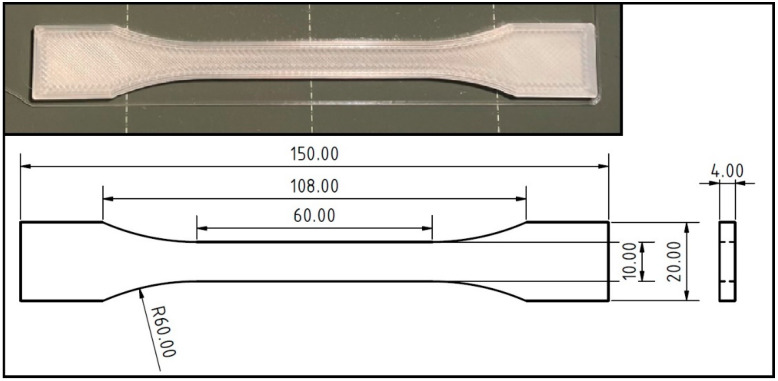
Shape and dimensions of the test specimen according to the ČSN EN ISO 527-2 standard corresponding to type 1B test specimens created by 3D printing.

A Prusa i3 MK3S (Prusa Research, a.s., Prague, Czech Republic) + 3D printer using FDM (fused deposition modeling) technology was used to print the standardized test specimens shown in Figure 2. The test specimens used in the research were designed and printed according to the ČSN EN ISO 527-2 standard and correspond to type 1B test specimens. The 3D printing parameters were based on the settings recommended by the supplier.

**Figure 3:**
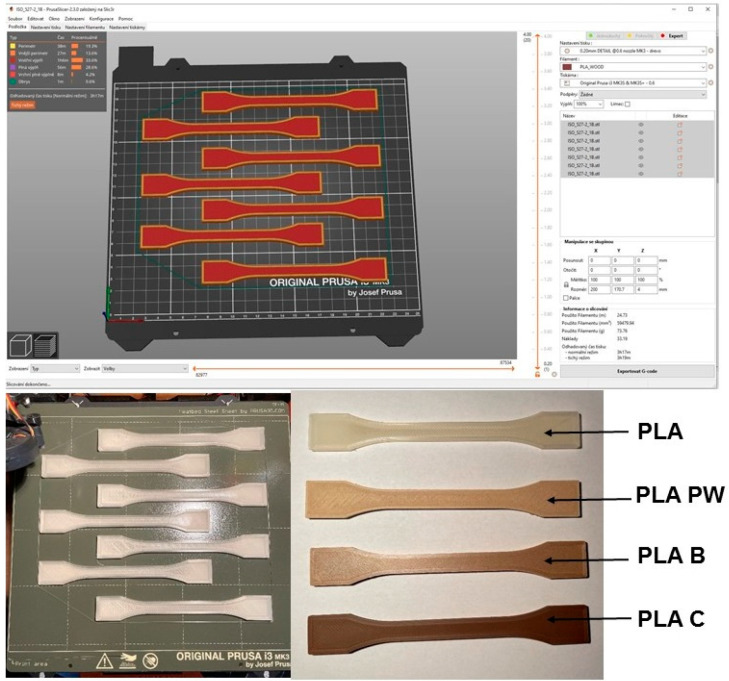
Three-dimensional printing kit and printed test specimens.

After unpacking, the test filaments were placed in a drying chamber at 55 °C for 6 h. A 0.6 mm diameter nozzle was used for printing to reduce the likelihood of clogging the nozzle. The layer height was chosen to be 0.2 mm. Three outer perms with 100% rectilinear fill at an angle of 45° and −45° to the *x*-axis were selected. The print speed was 45 mm × s^−1^ with a deceleration to 35 mm × s^−2^ on the outer perimeter. A speed of 20 mm × s^−1^ was used on the top solid filler to print the first layer. The printing nozzle temperature and the heated pad temperature were maintained at 200 °C and 50 °C, respectively. The cooling fan speed was set to 80% except for the first two layers where the cooling fan was turned off. The retraction distance was 5 mm, the retraction speed was 70 mm × s^−1^, and the detraction speed was 30 mm × s^−1^. During one printing cycle, 7 pieces of standardized test specimens were printed (Figure 3).

Printing was carried out from pure PLA and PLA material reinforced with PW, B, and C fillers under the same conditions.

### 2.2. Experiment Procedure

The fatigue life of test specimens created by 3D printing with FDM technologies from pure PLA and PLA reinforced with fillers based on pinewood (PW), bamboo (B) and cork (C) was investigated experimentally. All fatigue tests were performed on a universal test machine LABTest 5.50 ST (LABORTECH s.r.o., Opava, Czech Republic) with an AST KAF 50 kN measuring unit with Test & Motion software (LABORTECH s.r.o., Opava, Czech Republic) enabling cyclic tests with controlled voltage mode and load speed. Stress and strain data were recorded throughout the cyclic loading process. Cyclic tensile experiments on test samples were performed under controlled stress conditions at ambient room temperature. Experiments with uniaxial cyclic tensile stress were performed in the controlled stress mode using test specimens according to the ČSN EN ISO 527-2 standard corresponding to type 1B test specimens prepared by 3D printing using the FDM method. The test specimens always had the same shape and dimensions so that the results could be compared with each other. Jerez-Mesa et al. [39] found that the height of the layer has a significant effect on fatigue life in PLA. As the height of the PLA layer increased, better results were obtained in terms of the number of cycles to the failure of the test specimen.

During the static tensile test, the strength and deformation limits were determined for all the test specimens prepared by 3D printing from pure PLA, PLA PW, PLA B and PLA C at loading speed 0.6 mm × min^−1^. It was necessary to determine the reference values, which were subsequently used to set the test parameters of cyclic fatigue tests with 1000 cycles.

Low-cycle tests or quasi-static tests evaluate the service life of test specimens produced by 3D printing. The testing was conducted at different load intervals; 5–30%, 5–50% and 5–70% based on a simulation of a practical application in which the material is exposed to a lower to higher load approaching the maximum strength limit. This test interval for the load/force in the cyclic tests was based on the input values of the static reference value of the given test material, which differed significantly between the individual materials based on the PLA matrix.

**Figure 4:**
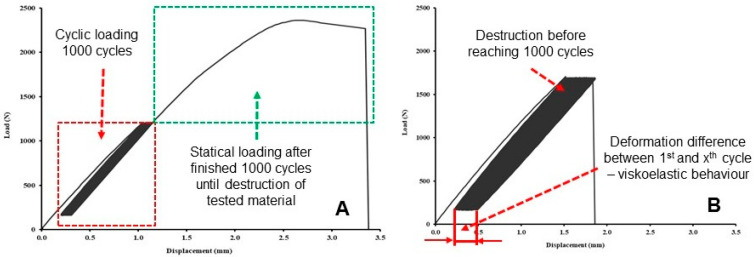
Load–displacement curves in the cyclic test: (**A**) satisfactory test—1000 cycles followed by destructive testing (PLA load in the interval 5 to 50%, 1000 cycles), (**B**) the tested material did not withstand 1000 cycles—premature termination of the destruction test (PLA load in the interval 5–70%, unfinished 1000 cycles).

Cyclic loading was carried out at 1000 cycles, with loading speed 0.6 mm × min^−1^ between the lower limit of the set reference value, i.e., 5% of the tensile static test, and 30%, 50% and 70% of the upper limit as given in Table 3. The endurance at the lower and upper limits was determined to be 0.5 s. The reason for the relatively long stay at the lower and upper limits within one cycle was based on the results of Zhang et al. [40], who stated that the short cycle time also reduces fatigue life. If the test sample lasted 1000 test cycles, the last load cycle was followed by the destruction of the test sample at the same test speed as in the static tensile test, with loading speed 0.6 mm × min^−1^ (Figure 4A). If the test specimens did not withstand cyclic fatigue testing, the test was terminated by premature destruction of the material and the number of cycles to failure was recorded (Figure 4B). The results of fatigue cyclic tests were the strength limit, deformation, and the difference in deformation between the 1st and 1000th cycles to determine viscoelastic behavior.

### 2.3. Structure Characterization

The fracture surface after the static and cyclic fatigue test was analyzed by scanning electron microscope (SEM) MIRA 3 TESCAN GMX SE (Tescan Brno s.r.o., Brno, Czech Republic) with an accelerating voltage of 5 to 15 kV and the Oxford SE detector (Tescan Brno s.r.o., Brno, Czech Republic). The surface of the tested samples was gilded with a Quorum Q150R ES—Sputtering Deposition Rate (Tescan Brno s.r.o., Brno, Czech Republic) using gold in an argon environment for SEM analysis.

## 3. Results and Discussion

**Figure 5:**
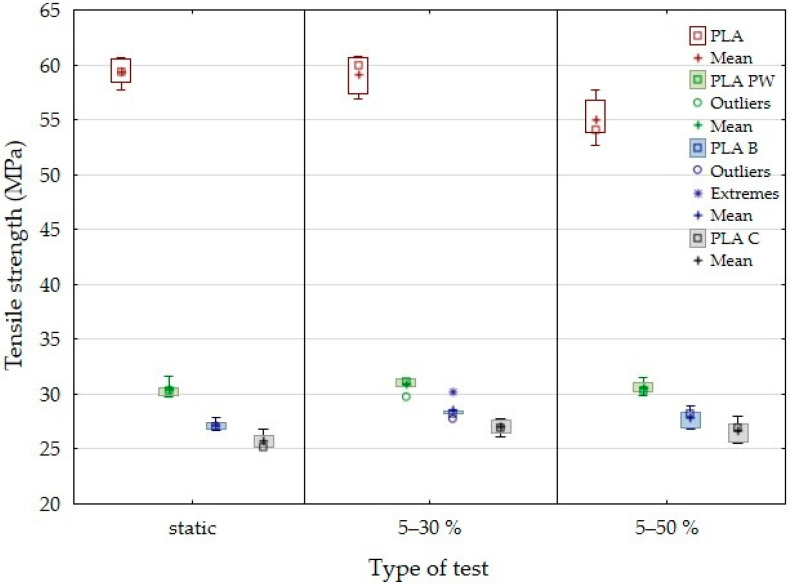
Tensile strength in static test and low-cycle test with load interval 5–30% and 5–50%.

**Figure 6:**
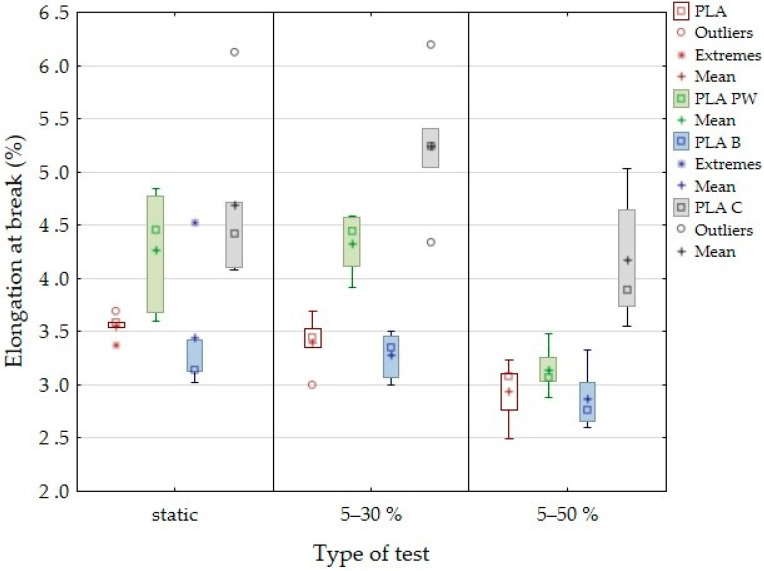
Deformation in static test and low-cycle test with load interval 5–30% and 5–50%.

The results of tensile strength and deformation in the static tensile test and low-cycle fatigue tests for the 3D printed samples from pure PLA and PLA reinforced with fillers based on pinewood (PW), bamboo (B) and cork (C) using FDM technology can be seen in Figure 5 and Figure 6, respectively. The results of the static tensile test confirmed the significant reduction of tensile strength in the particle-reinforced PLA samples (Figure 5). The tensile strength of pure PLA was 59.3 ± 1.2 MPa. With the addition of the biological fillers, there was a significant reduction in tensile strength. The results show that there was a significant decrease in tensile strength of pinewood (PW)-based sample by 48.7% (30.4 ± 0.7 MPa), bamboo (B)-based sample by 54.1% (27.2 ± 0.4%) and cork (C)-based PLA sample by 56.6% (25.7 ± 0.7 MPa). The results of the research are in line with other research dealing with similar issues. Kariz et al. [19] reported that as the proportion of wood in PLA increases, the strength decreases. Another example is Travieseo-Rodrigeuez et al. [28], who reported that cavities are formed between the wood-based filler and PLA that are more pronounced than in the case of a PLA without filler. These inconsistencies generally cause increased stresses within the material, which reduces the mechanical properties of PLA with filler [28]. The mechanical behavior of printed PLA with cork-based filler (15 wt.%) shows about 12% reduction in tensile strength compared to PLA [41]. However, it is clear that a more significant reduction in tensile strength was found in the experiments. Magalhães da Silva et al. [41] and Daver et al. [21] stated in their publications that PLA-based 3D-printed materials with cork exhibited reduced tensile strength and increased ductility. A similar trend of results was confirmed.

Arockiam et al. [31] stated in their research that the use of 3D printing materials in combination with bamboo fibers and polylactic acid (PLA) and polypropylene (PP) exhibit improved mechanical properties. The tensile strength was about 33 MPa. The tested material based on PLA with bamboo filler (PLA B), reached a tensile strength of about 27 MPa.

Research focusing on this issue consistently states that 3D-printed objects have characteristic positive aesthetic properties that are visible on the surface. The importance of the aesthetic properties of the final product based on biological filler, e.g., cork, is also shown by Gama et al. [42] in their research.

Figure 5 shows the tensile strength results and Figure 6 shows the deflection results for the low-cycle test with load intervals of 5 to 30% and 5 to 50%. In this case, the test samples withstood 1000 test cycles (Table 4). 

Cyclic testing did not affect the change of tensile strength and associated durability over this load interval for PLA-based biological filler-reinforced materials. PLA PW showed a slight increase in tensile strength of 1.6%, while PLA B and PLA C showed an increase in tensile strength up to 5%. In Table 1 and Figure 1, it can be seen that PLA C showed the smallest filler size of the materials tested. The average size was approximately 12 µm. The positive effect of smaller particles influencing fatigue life has been mentioned in previous publications. The results of the research focused on the cyclic fatigue of the tested materials using 3D printing technology in manufacturing to demonstrate an increased fatigue life using biological fillers in PLA filament. Shahar et al. [36] and Abdullah et al. [43] reported an increased fatigue life in the case of the addition of fillers/particles of kenaf. They reported that the probable reason was that the fine particles of kenaf powder were able to form a good interfacial bond with the PLA matrix and better stress distribution in the matrix [36]. Determining the highest proportion of cycles to failure at different load values is very important and can be seen in Table 4. 

The fine particles have a larger surface area that interacts with the PLA matrix, thus increasing the contact or interface between the filler and the matrix. Stress transfer becomes more efficient with finer filler particles in the matrix. This is because the stress applied to the matrix is evenly distributed to the individual particles at the interface [36].

During filament preparation and subsequent additive manufacturing, high-temperature processing results in rapid diffusion, causing interdiffusion bonding between the biological filler (powder) and PLA. Good contact between the filler and the PLA could improve the resulting mechanical properties [36].

From a practical application point of view, it is essential to find that cyclic fatigue up to 50% of the load does not reduce the tensile strength. For PLA, the tensile strength decreased by 7.2% with a cyclic load in the range of 5 to 50%. The results revealed that pure PLA and PLA material reinforced with biological fillers were not able to complete 1000 test cycles with a load interval of 5–70% (Table 4).

It can be seen in Figure 6 that the PLA showed a gradual decrease in deformation under cyclic loading, from 3.6% in the static test to 2.9% in the low-cycle fatigue test in the load interval between 5% and 50%. PLA B material showed a similar trend, i.e., deformation decreased from 3.5% found in static tests to 2.9% in the low-cycle fatigue test in the load interval between 5% and 50%. A similar trend of decreasing deformation due to cyclic fatigue was determined for PLA PW. For the tested PLA C materials, there was an increase in deformation values due to the cyclic load in the interval of 5 to 30% and only subsequently a decrease in the cyclic load in the interval of 5 to 50%. Deformation in a low-cycle test is very important and can predict subsequent failure, i.e., material destruction. The deformation difference seen in Figure 6 is an important factor.

**Figure 7:**
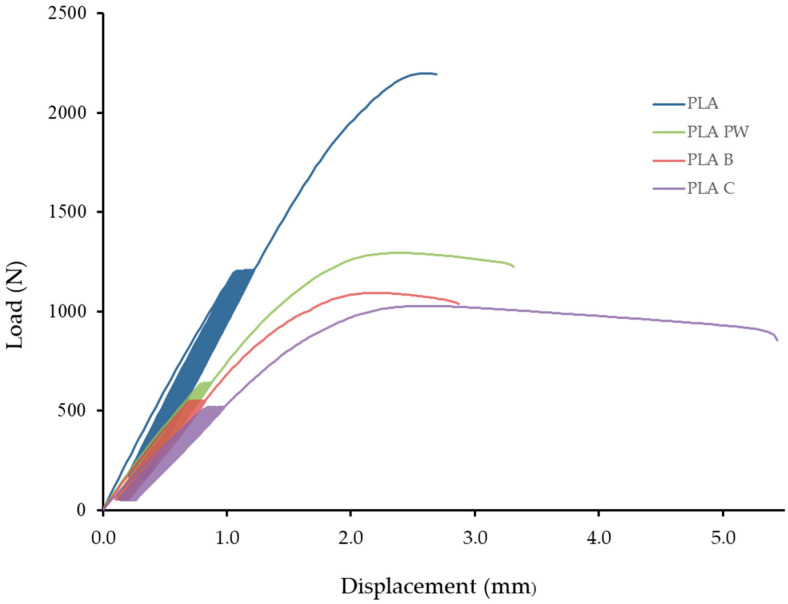
Low-cycle fatigue behavior of 3D-printed PLA reinforced with natural reinforcement PW, B and C at loads in the range 5–50%—PLA (121 to 729 N), PLA PW (64 to 383 N), PLA B (55 to 330 N), PLA C (55 to 310 N).

Figure 7 shows an example of low-cycle fatigue behavior of 3D-printed PLA reinforced with natural reinforcements PW, B, and C. It can be seen that 1000 cycles were first performed and then immediately last cycle and failure test. Figure 7 shows a low-cycle load from which the difference in deformation between the 1st and 1000th cycles is apparent. The difference in deformation represents the difference between the deformation after the 1st and 1000th cycle. The difference in deformation depends on the load and elongation. This value affects the overall load profile and the resulting mechanical properties. The different height of the hysteresis loop represents a different value of the force set in the range between 5% and 50%, resulting from a different value of the force resulting from the static tensile test.

Figure 7 shows the viscoelastic behavior (creep) of the tested materials at a cyclic load of 5 to 50%. Similar behavior was found for cyclic loads of 5 to 30% and 5 to 70%. It clearly shows the constant elongation during cyclic loading corresponding to the constant fatigue of the tested materials created using 3D printing technology.

It is also clear from the results that the longer the relative deformation after the last cycle, the sooner the destruction will occur, and the tested materials will not withstand the given number of 1000 cycles.

The maximum and minimum deformations of each cycle are plotted in Figure 7. From the figures it is not clear that load and displacement loops tend to be slimmer with increasing cycles and more linear as with the epoxy polymeric material reported in the literature [37].

The tested samples showed cyclic creep, during which there is an accumulation of plastic deformation due to the cyclic mechanical stress manifested by plastic deformation characterized by the displacement of hysteresis loops of stress and strain, which is evident in Figure 7 and Table 4 [44].

If cyclical stress exceeds the elastic limit, there is an accumulation of plastic deformation, which is then the cause of destruction of the test specimen [45]. This condition occurred at a load of 5 to 70%. Table 4 shows a significant increase in relative deformation after last cycle at loads in the range of 5 to 70% compared to other cyclic tests at lower load values. Relative deformation after the last cycle ranged from 0.52 to 1.85%.

Zhang et al. [40] found in adhesive bond research that an increase in cyclic amplitude stress can reduce fatigue life at low cycles.

However, for epoxy resins, Gang et al. [45] have found that this cyclic ratcheting is a renewable viscoelastic deformation that does not cause damage to the material. This effect was demonstrated for results at loads of 5 to 30% and 5 to 50%.

The effect of hysteresis loops seen in Figure 7 is very important in the formation of permanent deformation in products caused by their cyclic loading. This permanent deformation is presented by the results of relative deformation after finishing the 1st cycle and relative deformation after the last cycle given in Table 4, from which it is clear that with increasing values of cyclic loading up to 30%, 50% and 70%, permanent deformation of the tested materials also increases.

Due to cyclic loading, a complex stress state was created in the material, caused mainly by the tensile stress. After releasing this stress, the test specimen without load tended to return to its original state. However, the accumulated stress and the associated deformation led to viscoelastic behavior, i.e., a shift in the hysteresis loop, as can be seen in Figure 7 and Table 4.

Senatov et al. [46] stated that the displacement of the hysteresis loop makes it possible to evaluate the value of the accumulated deformation for the respective cycle, or the number of cycles. Change to the width and area of the hysteresis loop allows us to evaluate the value of the reversible deformation that was dissipated during the cyclic loading [46].

**Figure 8:**
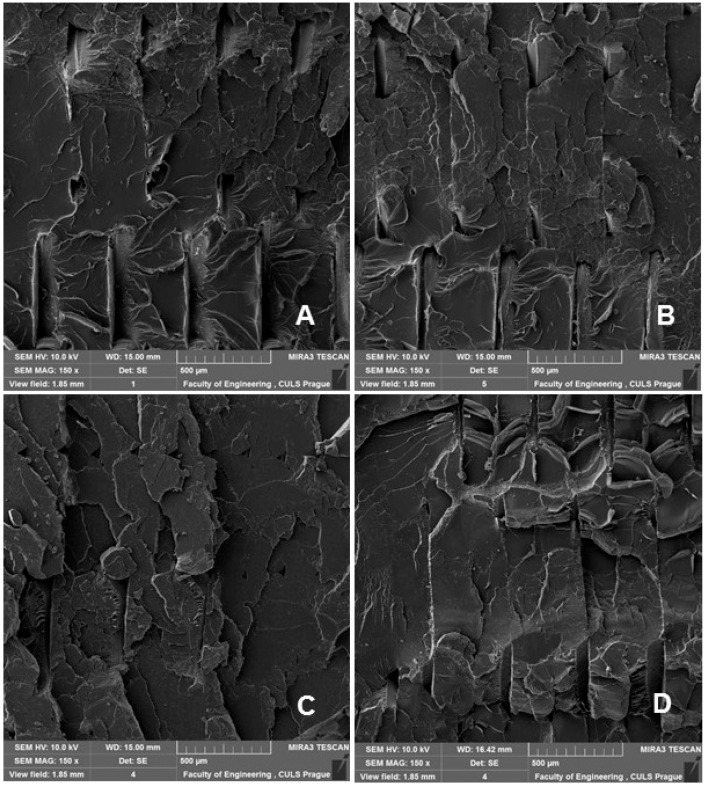
SEM images and fractography analysis of 3D-printed material PLA (MAG 150×): (**A**) Static tensile test, (**B**) Load in cyclic test 5 to 30%, (**C**) Load in cyclic test 5 to 50%, (**D**) Load in cyclic test 5% up to 70%.

**Figure 9:**
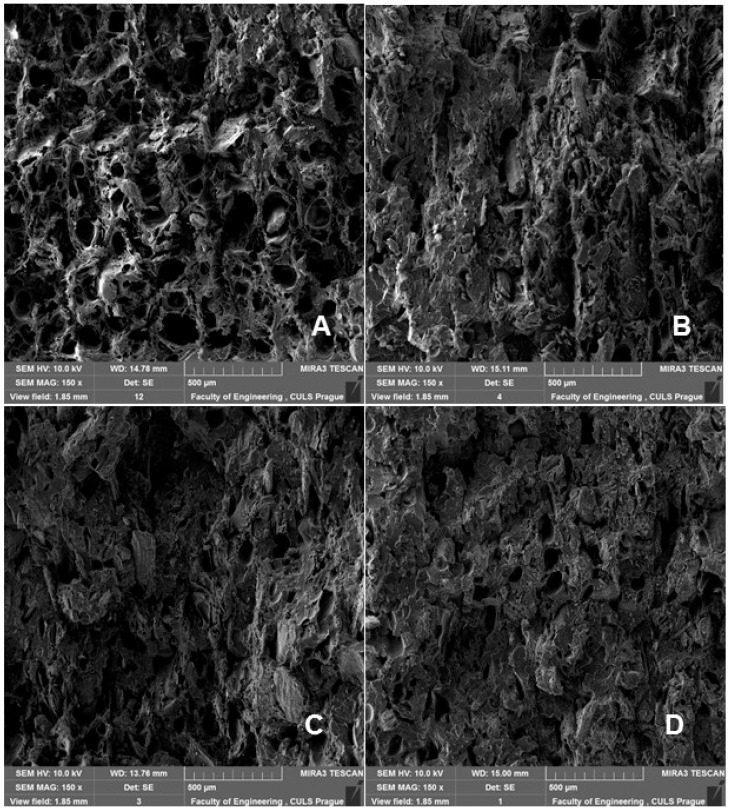
SEM images and fractography analysis of 3D-printed material PLA PW (MAG 150×): (**A**) Static tensile test, (**B**) Load in cyclic test 5 to 30%, (**C**) Load in cyclic test 5 to 50%, (**D**) Load in cyclic test 5 to 70%.

**Figure 10:**
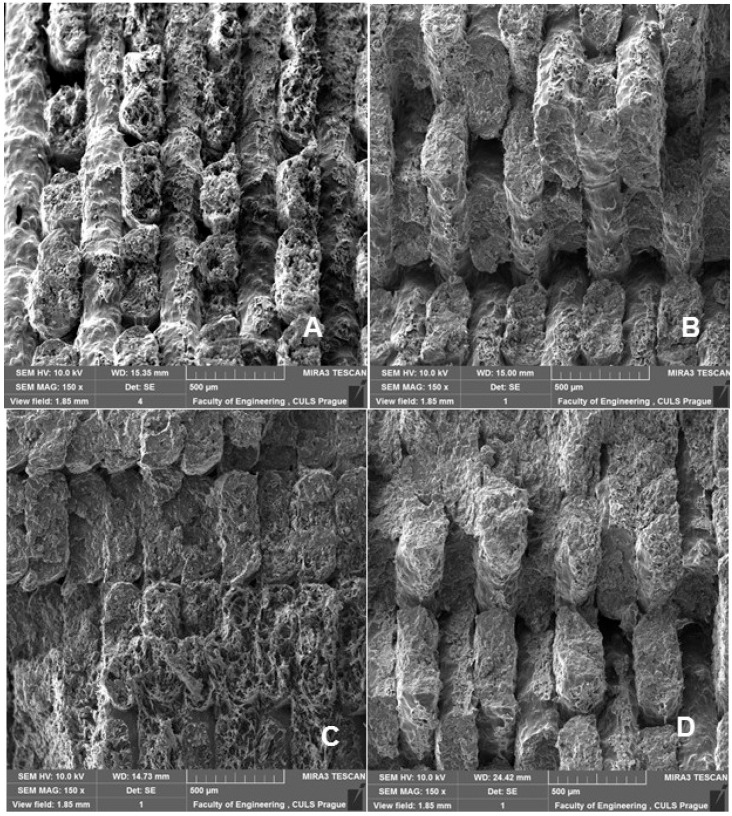
SEM images and fractography analysis of 3D-printed material PLA C (MAG 150×): (**A**) Static tensile test, (**B**) Load in cyclic test 5 to 30%, (**C**) Load in cyclic test 5 to 50%, (**D**) Load in cyclic test 5 to 70%.

**Figure 11:**
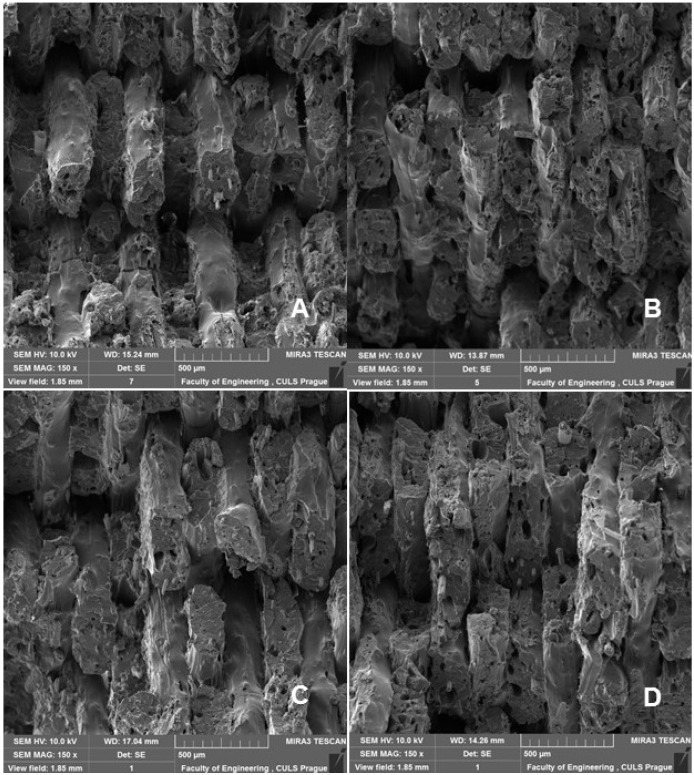
SEM images and fractography analysis of PLA B 3D-printed material (MAG 150×): (**A**) Static tensile test, (**B**) Load in cyclic test 5 to 30%, (**C**) Load in cyclic test 5 to 50%, (**D**) Load in cyclic test 5 to 70%.

SEM analysis is therefore an important and universal tool for checking the structure of the tested materials and, in the case of a material based on a composite structure, the interface between the filler and the matrix. SEM images of the fracture surface of 3D-printed test specimens after destructive testing are shown in Figure 8, Figure 9, Figure 10 and Figure 11. Figure 8 shows the brittle fracture surface of the PLA material without filler. Figure 8 shows a smooth fracture surface of pure PLA.

Figure 9, Figure 10 and Figure 11 show regular and irregular elongated cavities/pores. Azadi et al. [6] stated that, in 3D printed PLA, there are two categories of porosity, which arise from production (relatively small, regular spherical shape) and inconsistent connection of individual layers, which are manifested by irregular slit-shaped cavities.

Yadollahi et al. [47] stated that the porosity attributed to production may be due to nonideal fiber formation during production. This type of porosity occurred in the tested materials with biological filler, i.e., PLA PW, PLA B, PLA C. This porosity has the effect of reducing the mechanical properties and reducing the density of the 3D printed test specimens. This conclusion was verified by measuring the weight of the test specimens on highly sensitive KERN analytical balances. The weight of the test specimen made by 3D printing from PLA was 10.11 ± 0.05 g, PLA PW 9.60 ± 0.16 g, PLA B 9.61 ± 0.27 g and PLA C 9.68 ± 0.17 g. A comparative test was performed with the injection molded PLA filament in a BOY injection molding machine with a mold corresponding to the test specimen, resulting in a weight of 10.28 ± 0.04 g. This comparison was made because commercial thermoplastic products are fabricated primarily by injection molding. It is clear from the results that with the addition of the filler, the weight of the 3D-printed test specimens PLA WP, PLA B and PLA C decreased in the range from 4.3 to 5%. Gama et al. [42] stated that the weight of products made by 3D printing was reduced by adding cork.

**Figure 12:**
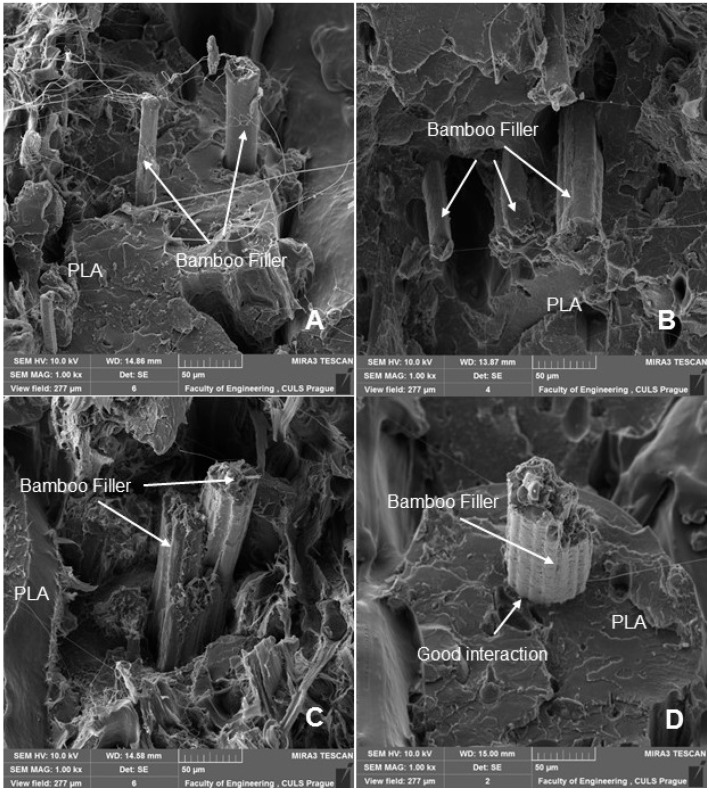
SEM images and fractography analysis of PLA B 3D-printed material (MAG 1000×): (**A**) Static tensile test, (**B**) Load in cyclic test 5 to 30%, (**C**) Load in cyclic test 5 to 50%, (**D**) Load in cyclic test 5 to 70%.

**Figure 13:**
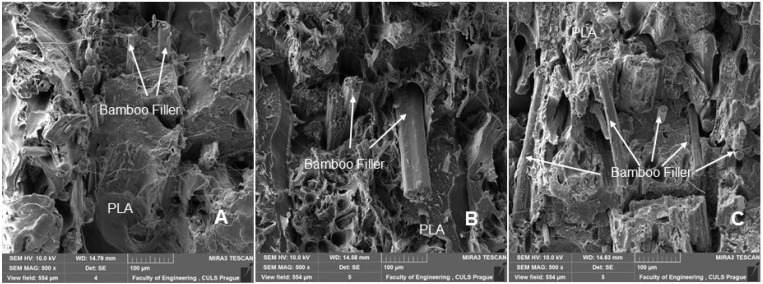
SEM image and fractography analysis of PLA B 3D-printed material (MAG 500×): (**A**) Static tensile test, (**B**) Load in cyclic test 5 to 50%, (**C**) Load in cyclic test 5 to 70%.

**Figure 14:**
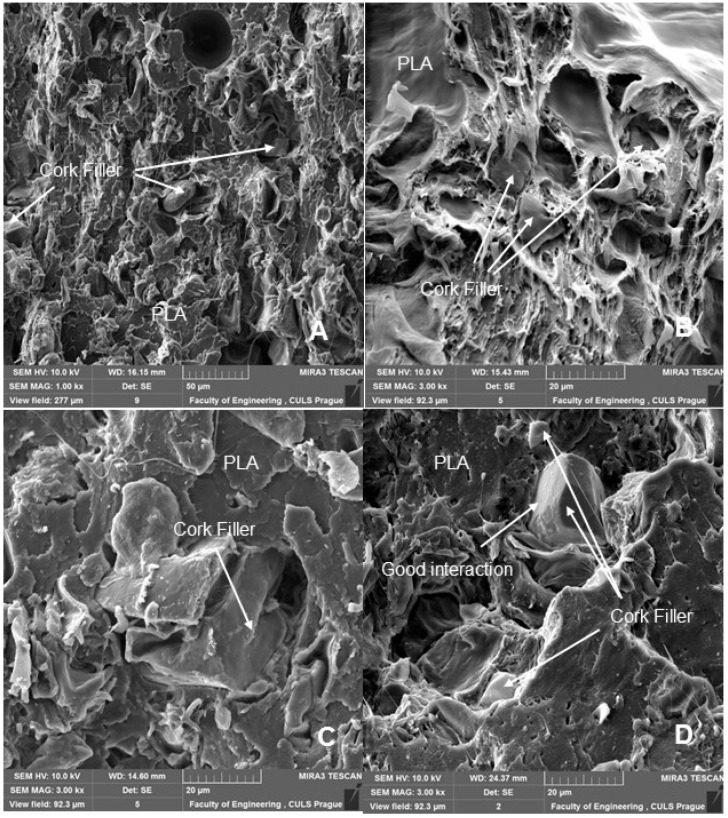
SEM images and fractography analysis of 3D-printed material PLA C: (**A**) Static tensile test (MAG 1000×), (**B**) Static tensile test (MAG 3000×), (**C**) Cyclic test load 5 to 50% (MAG 3000×), (**D**) Cyclic test load 5 to 70% (MAG 3000×).

Fractographic analysis (Figure 8, Figure 9, Figure 10 and Figure 11) of the transverse fracture of test specimens at different cyclic loads could provide more information on the failure mode of 3D printed pure PLA and PLA samples reinforced with pinewood (PW), bamboo (B) and cork (C)-based fillers. The air bubbles, the arrangement of the printing layers, and the interaction of the PLA with the filler can be seen in Figure 9, Figure 10, Figure 11, Figure 12, Figure 13 and Figure 14.

**Figure 15:**
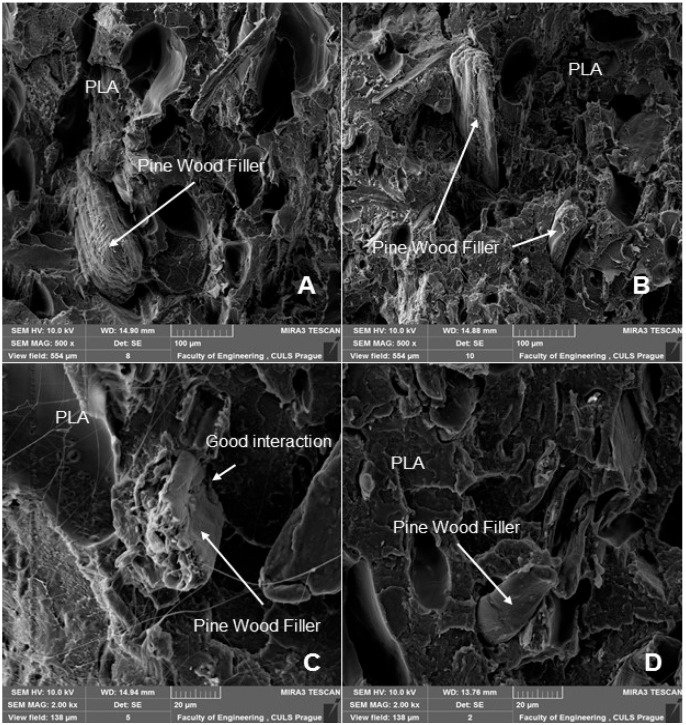
SEM images and fractography analysis of 3D-printed material PLA WP: (**A**) Load in cyclic test 5 to 50% (MAG 500×), (**B**) Load in cyclic test 5 to 70% (MAG 500×), (**C**) Static tensile test (MAG 2000×), (**D**) Load during cyclic test 5 to 50% (MAG 2000×).

The SEM images with a higher magnification of MAG 500× to 3000× show the fracture surface of the test specimens, the geometric shape of the PW, B and C filler and its mutual interaction with the PLA matrix. Figure 12, Figure 13, Figure 14 and Figure 15 show the propagation of the crack on several surfaces with different height levels shifted relative to each other. The quarry areas were contiguous and interconnected or separated. Generally, in PLA-based composites with biological fillers, there are more irregular and rougher fracture surfaces [7].

A natural defect in products made by 3D printing is the occurrence of interlayer disorders [27]. The results showed that the interfacial compatibility of PLA with the filler is generally good.

Based on the SEM analysis, it has not been proven that wood particles have any detrimental effect on the PLA matrix because it reduces the cohesion of the deposited fibers [28]. However, the tensile strength dropped significantly against pure PLA without filler.

By using polymeric materials with natural fillers from waste (e.g., wood, bamboo or cork), the cost of raw materials can be reduced. At the same time, the environmental burden is reduced by reducing the consumption of the input polymer by adding a biodegradable filler in PLA. Overall, the resulting composite remains biodegradable. Another important factor is the aesthetic potential of these materials, which is pointed out by various scientific works. The potential use of PLA material in combination with natural fillers can be in decoration, furniture, and the automotive industry. The relatively lower density of the cellulose-based filler may lead to an overall lower density of the resulting materials. This is desirable in certain applications if sufficient mechanical performance is maintained for the type of application. Three-dimensional printing can also produce complex geometry while maintaining a low weight, for example, for sound insulation. Santos Silva et al. [48] investigated the dynamic properties of a set of sandwich panels with cork-based cores, and the results showed that a cork mixture can be used as an effective passive damping material in laminated or sandwich constructions.

Filament development based on PLA and cork represents an ideal color modification for transparent material without the need to add synthetic dyes [41]. Reinforced with wood and other natural fillers, PLA materials have been developed for 3D printing to offer consumers a way to create products that resemble wooden parts and at the same time offer enhanced durability [28]. From the above, the need for intensive research activities in this area is clear.

## 4. Conclusions

This paper presents the results of low-cycle fatigue behavior of 3D-printed pure PLA and PLA reinforced with natural reinforcements e.g., pinewood, bamboo and cork. From the results of static tensile testing, it was observed that there is a reduction in tensile strength due to the addition of biological fillers ranging from about 49 to 57% with respect to PLA without filler. The static tensile strength increased by about 31% for cork filler-reinforced PLA and by about 19.5% for pinewood-based PLA. There was a slight decrease in ductility of about 3% when using bamboo-based filler.

Different loading intervals, e.g., 5–30%, 5–50% and 5–70%, were used in this research based on the simulation of a practical application where the material is subjected to a lower to higher load close to the maximum strength. This cyclic loading test interval was based on the input values of the static test, which varied significantly between the various PLA-based materials.

The cyclic fatigue tests of 3D-printed pure PLA and PLA reinforced with natural fillers from pinewood, bamboo and cork were performed under controlled loading mode using different combinations of load intervals, e.g., 5–30%, 5–50% and 5–70% based on the input values of the static tests. Quantitative conclusions were derived from the stress and strain behavior. Comparison of the cyclic fatigue test results for pure PLA and PLA reinforced with pinewood, bamboo and cork fillers showed that under the same loading conditions, the fatigue life of the 3D printed specimens was similar. The fillers did not have any significant effect on the change to tensile strength and associated durability over this loading intervals for PLA-based materials reinforced with biological fillers.

From a practical application point of view, it is essential to note that cyclic fatigue up to 50% of the load specified for the material in the static test does not reduce the tensile fatigue strength. This was found by testing the pure PLA and PLA reinforced with biological filler materials. Both types of samples were unable to pass 1000 test cycles with a load range of 5 to 70%. Under cyclic loading, the viscoelastic behavior of the tested materials was determined from the results of relative deformation after finishing the 1st cycle and relative deformation after the last cycle. From the results, it was clear that as the cyclic loading value of 30%, 50% and 70% increases, the permanent deformation of the tested materials, i.e., viscoelastic behavior (creep), also increases. Results of the low-cycle fatigue test for 3D-printed PLA reinforced with pinewood, bamboo and cork fillers showed that the addition of filler at different cyclic loading intensity reduced the relative deformation after the last cycle with respect to PLA without filler. The ratcheting deformation accumulated during cyclic loading under controlled stress has essentially no detrimental effect on the fatigue life of the tested materials.

Research has confirmed that the use of PLA reinforced with natural fillers e.g., pinewood, bamboo, and cork is feasible, especially in terms of cyclic fatigue tests.

SEM analysis showed the presence of interlayer defects in the fracture of the tested materials that are directly related to the 3D printing manufacturing technology. At the same time, very good interfacial compatibility of PLA with the biological fillers was established.

## Figures and Tables

**Table 1:** Measurement of PLA reinforced with natural reinforcement in a direction perpendicular to the cross section.

PLA Reinforced with Natural Reinforcement	Bamboo (B)		Pinewood (PW)		Cork (C)	
	Dimension A	Dimension B	Dimension A	Dimension B	Dimension A	Dimension B
Arithmetic mean (µm)	22.9	18.5	77.6	31.4	12.7	11.2
Deviation (µm)	12.6	9.1	61.0	25.0	6.9	7.7
Coefficient of variation (%)	55.3	48.9	78.6	79.8	54.1	68.3

**Table 2:** Basic parameters for 3D printing according to the filament supplier’s recommendations [38].

Designation	Dimension [mm]	Hot End Temperature [°C]	Build Surface Temperature [°C]	Cooling Fan [%]	Working Temperature Range [°C]	Printing Speed [mm × s^−1^]
PLA PW	1.75 ± 0.05	195–225	20–60	20–50	0–60	40–120
PLA B	1.75 ± 0.05	195–225	20–60	20–50	0–60	40–120
PLA C	1.75 ± 0.05	195–225	20–60	20–50	0–60	40–120
PLA	1.75 ± 0.05	195–225	20–60	20–50	0–60	40–120

**Table 3:** Input load parameters during cyclic fatigue tests.

Material	Load in the Interval 5–30%	Load in the Interval 5–50%	Load in the Interval 5–70%
PLA	121 to 729 N	121 to 1215 N	121 to 1701 N
PLA PW	64 to 383 N	64 to 638 N	64 to 894 N
PLA B	55 to 330 N	55 to 549 N	55 to 769 N
PLA C	55 to 310 N	52 to 517 N	52 to 723 N

**Table 4:** Results Low-cycle fatigue behavior of 3D printed PLA reinforced with natural reinforcement PW, B and C.

3D Printed Material	Low-Cycle Test	Number of Cycles	Number of Test Samples (Number of Finished Cycles/Total Number of Tests)	Relative Deformation after Finishing 1st Cycle	Relative Deformation after Last Cycle
PLA	from 5 % to 30 % (121 to 729 N)	1000 ± 0	5/5	0.15 ± 0.00%	0.19 ± 0.01 %
	from 5 % to 50 % (121 to 1215 N)	1000 ± 0	5/5	0.19 ± 0.01%	0.32 ± 0.02 %
	from 5 % to 70 % (121 to 1701 N)	438 ± 53	0/5	0.23 ± 0.02%	0.52 ± 0.07 %
PLA PW	from 5 % to 30 % (64 to 383 N)	1000 ± 0	5/5	0.09 ± 0.00%	0.12 ± 0.00 %
	from 5 % to 50 % (64 to 638 N)	1000 ± 0	5/5	0.11 ± 0.01%	0.19 ± 0.01 %
	from 5 % to 70 % (64 to 894 N)	643 ± 91	0/5	0.14 ± 0.00%	0.65 ± 0.09 %
PLA B	from 5 % to 30 % (55 to 330 N)	1000 ± 0	5/5	0.07 ± 0.01%	0.10 ± 0.01 %
	from 5 % to 50 % (55 to 549 N)	1000 ± 0	5/5	0.09 ± 0.00%	0.16 ± 0.02 %
	from 5 % to 70 % (55 to 769 N)	727 ± 72	0/5	0.12 ± 0.01%	0.63 ± 0.15 %
PLA C	from 5 % to 30 % (55 to 310 N)	1000 ± 0	5/5	0.09 ± 0.00%	0.14 ± 0.01 %
	from 5 % to 50 % (52 to 517 N)	1000 ± 0	5/5	0.11 ± 0.01%	0.23 ± 0.01 %
	from 5 % to 70 % (52 to 723 N)	677 ± 154	0/5	0.15 ± 0.01%	1.85 ± 0.35 %

## Data Availability

Not applicable.
